# Mediators of Yoga and Stretching for Chronic Low Back Pain

**DOI:** 10.1155/2013/130818

**Published:** 2013-04-17

**Authors:** Karen J. Sherman, Robert D. Wellman, Andrea J. Cook, Daniel C. Cherkin, Rachel M. Ceballos

**Affiliations:** ^1^Group Health Research Institute, 1730 Minor Avenue, Suite 1600, Seattle, WA 98101, USA; ^2^Department of Epidemiology, University of Washington, Seattle, WA 98101, USA; ^3^Department of Biostatistics, University of Washington, Seattle, WA 98101, USA; ^4^Departments of Family Medicine and Health Services, University of Washington, Seattle, WA 98101, USA; ^5^Fred Hutchinson Cancer Research Center, 1100 Fairview Avenue North, Seattle, WA 98109, USA

## Abstract

Although yoga is an effective treatment for chronic low back pain, little is known about the mechanisms responsible for its benefits. In a trial comparing yoga to intensive stretching and self-care, we explored whether physical (hours of back exercise/week), cognitive (fear avoidance, body awareness, and self-efficacy), affective (psychological distress, perceived stress, positive states of mind, and sleep), and physiological factors (cortisol, DHEA) mediated the effects of yoga or stretching on back-related dysfunction (Roland-Morris Disability Scale (RDQ)). For yoga, 36% of the effect on 12-week RDQ was mediated by increased self-efficacy, 18% by sleep disturbance, 9% by hours of back exercise, and 61% by the best combination of all possible mediators (6 mediators). For stretching, 23% of the effect was mediated by increased self-efficacy, 14% by days of back exercise, and 50% by the best combination of all possible mediators (7 mediators). In open-ended questions, ≥20% of participants noted the following treatment benefits: learning new exercises (both groups), relaxation, increased awareness, and the benefits of breathing (yoga), benefits of regular practice (stretching). Although both self-efficacy and hours of back exercise were the strongest mediators for each intervention, compared to self-care, qualitative data suggest that they may exert their benefits through partially distinct mechanisms.

## 1. Introduction

**Figure 1:**
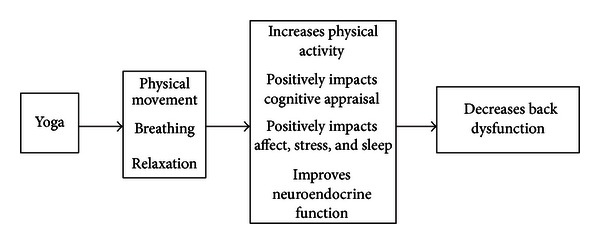
Model describing possible mechanisms underlying the effectiveness of yoga for chronic low back pain.

In the last decade, results from 11 clinical trials evaluating yoga as a treatment for chronic back pain have been published [1–11]. Most had substantial limitations [1, 2, 4, 6–8], including small studies with large baseline imbalances on baseline back pain, back dysfunction or key descriptors of back pain history [1, 2, 4], low class attendance [7], and poor follow-up rates [6, 8]. Nonetheless, all of the trials suggest that yoga is beneficial for back pain. However, few studies have collected data designed to shed light on the mechanisms responsible for yoga's benefits. Yoga includes physical movements, but it is a complex intervention involving other components such as specialized use of the breath and relaxation [12]. A slightly modified version of Sherman et al.'s [13] heuristic model describing plausible mechanisms that could act synergistically to produce yoga's benefits is presented here (Figure 1). Broadly, these mechanisms include improvements in physical functioning of the back, cognitive appraisal about back pain, and general affect, stress, sleep, and neuroendocrine function. Improved physical function could result from improvements in spinal flexibility [1, 5, 7], increased hamstring flexibility [14], and increased strength and flexibility [15]. Cognitive appraisal could include fear avoidance [16], improvements in self-efficacy [17, 18], and improvements in body awareness [19, 20]. General affect and stress includes improved mood and subjective well-being and reduced anxiety and depression [12, 21]. In addition, the model hypothesizes that improved neuroendocrine function is associated with improvements in affect and stress.

As part of a large trial of yoga compared with stretching exercise of comparable physical exertion and usual care [9], we collected data on the most relevant potential mediators of treatment to explore possible mechanisms by which yoga might exert its benefits. We have reported that yoga and conventional stretching exercises had similar benefits but were superior to self-care [9]. This report presents the mediator analyses comparing yoga to self-care and stretching to self-care. We test the hypothesis that baseline to 6-week changes in the measures of physical activity, cognitive appraisal, general affect and stress, and neuroendocrine function mediate the effects of yoga and stretching on changes in back-related dysfunction over the 12-week intervention. Qualitative findings from the yoga and exercise groups place the quantitative findings in a broader context.

## 2. Methods

### 2.1. Population, Recruitment, and Procedures

Because our trial protocol [13] and primary results [9] have been reported elsewhere, the trial is described only briefly here. The study was conducted in six locations in the Puget Sound region of Washington state, USA. Most participants were recruited from an integrated healthcare system and all had nonspecific low back pain for at least 3 months, were between 20 and 64 years, and rated their pain at least 3 on an 11-point scale. We excluded individuals who had back pain of a known (e.g., vertebral fracture) or complex (e.g., sciatica) cause, were seeking compensation, had comorbid conditions precluding clear interpretation of findings (e.g, fibromyalgia), were unwilling or unable to attend class, or unable to give fully informed consent.

A total of 228 participants were randomized in a 2 : 2 : 1 ratio to 12 weeks of weekly yoga classes, 12 weeks of weekly intensive stretching classes, or a self-care book. Participants were permitted to seek additional health care as needed, but this was uncommon and did not differ between groups. Follow-up interviews by interviewers masked to treatment were conducted by telephone at 6, 12, and 26 weeks after randomization.

Attendance at classes was similar in both yoga and stretching, with 65% of the yoga participants versus 59% of the stretching participants attending at least 8 classes. Home practice was also comparable. For example, in the last week of classes, 71% of yoga class attendees versus 63% of stretching class attendees reported practicing at least 3 times at home, with a median of 20 minutes per class in both groups.

Because mediation is the focus of this analysis, we included only the 192 (84% of original) participants who had both 6- and 12-week follow-up data (78 in yoga, 74 in stretching, and 40 in usual care). Of those, we had saliva data for 133 participants (57 in yoga, 51 in stretching, and 25 in self-care).

### 2.2. Measures

#### 2.2.1. Overview

Mediating variables are those responsible for all or part of the effects of a treatment on outcome. Therefore, a mediator variable must change during treatment, be associated with treatment, and have an effect on outcome. In this trial, most of our mediator variables were specified in our heuristic model and developed prior to the funding of the study. Sleep disturbance was added because it is known to have an important effect on back pain. Hours of back exercise were added as a proxy for physical movement.

#### 2.2.2. Potential Mediating Variables

We measured variables representing a number of aspects of cognitive appraisal, affect and sleep, physical movement, and neuroendocrine function that we hypothesized would mediate any effects of yoga on low back pain. 

*(1) Cognitive Appraisal*. Aspects of cognitive appraisal were assessed with measures of fear avoidance, self-efficacy, and self-awareness. 

Fear avoidance was measured with the Tampa Scale for Kinesiophobia [22], a 17-item scale measuring back pain patients' fears of movement, exercise, and serious underlying disease. We used 10 of the 17 items from this scale, eliminating several items found highly redundant or confusing to participants [23]. This 10-item version retains acceptable internal consistency (alpha = 0.76), is easier and quicker to administer, and has proved sensitive in detecting intervention effects in clinical trials [23].

Self-efficacy was measured with a 5-item version of the validated and reliable Arthritis Self-Efficacy Scale [24] modified for back pain patients. 

Awareness was measured specifically as conscious awareness of the body using two complementary validated questionnaires, the Body Awareness Questionnaire [25] and the Body Responsiveness Questionnaire [20]. The Body Awareness Questionnaire contains 18 items that measure self-reported attentiveness to normal nonemotive bodily processes, including sensitivity to body cycles and rhythms, ability to detect small changes in normal function, and ability to anticipate bodily reactions. It has been found to have good internal consistency and test-retest reliability [25]. The Body Responsiveness Questionnaire, a 7-item scale designed to measure responsiveness to bodily sensations, also has good internal consistency (Cronbach's alpha = 0.83) [20]. However, many of the questions in these scales were unlikely to measure relevant outcomes of enhanced body awareness for students of yoga (e.g., I can always tell when I bump myself whether or not it will become a bruise; I notice specific body reactions to the weather). We therefore asked seven experienced yoga teachers from around the US to review these 25 questions and independently rate the likelihood that each question would capture a construct likely to change with yoga. For our mediator analyses, we selected the 8 questions that most yoga teachers thought would be likely to change (e.g., I “listen” to my body to advise me what to do; I enjoy becoming aware of how my body feels).

*(2) Affect and Stress*. We measured psychological distress, perceived stress, and positive states of mind. In addition, we measured impact of back pain on sleep disturbance, which was not part of the original model.

Psychological distress was measured with the 5-item mental health index of the SF-36 [26]. This scale, which assesses general mental health, including depression, anxiety, behavioral-emotional control, and general positive affect, is brief and reliable and has shown good agreement with more comprehensive measures of mental health [27].

Perceived stress was measured with the 10-item version of the perceived stress scale [28], the most widely used self-report measure of psychological stress. 

Positive states of mind were measured with the positive states of mind scale, a 6-item scale that has good internal consistency (Cronbach's alpha ranges from 0.65 to 0.77) [29, 30] and is inversely related to anxiety and to indicators of stress [30].

Sleep quality was measured using one question from the Roland-Morris Disability Index [31]. The question “I sleep less well because of my back” is answered as Yes or No.

*(3) Physical Activity*. To remove physical activity as a mediator, we measured hours of back exercise by asking about the number of hours the participant had performed back exercises in the past week.

*(4) Physiological Measures of Neuroendocrine Function*. We measured *cortisol* and *DHEA* from saliva samples. Both measures are highly correlated with serum levels [32, 33]. Saliva samples were collected from each participant immediately upon waking, 30 minutes after waking, and at bedtime over a two-day period (6 samples total) at baseline, 6 and 12 weeks. Prior to collecting saliva samples, participants were asked not to brush their teeth, use alcohol (in prior 12 hours), eat or smoke (within prior hour), or take corticosteroids. Cortisol levels were measured using all 6 samples, while DHEA was measured only at waking and bedtime. Mean values were calculated for each time point. The saliva samples were analyzed using enzyme immunoassays for cortisol and DHEA at the STAR (Saliva Testing and Reference) Laboratory, Seattle, WA, USA, a CLIA certified laboratory specializing in the analysis of salivary samples. Immunoassays were conducted using commercially available kits purchased from Salimetrics (State College, PA, USA). 

#### 2.2.3. Primary Outcome Measure

The primary outcome measure was the modified 23-item Roland-Morris Disability Questionnaire (RDQ), a measure of back-related patient dysfunction [31]. It is one of the two most popular instruments used by back pain researchers for measuring function [34], takes approximately five minutes to complete [35], has been found to be reliable, valid, and sensitive to clinical changes [31, 36–40], and is well suited for telephone administration [34].

### 2.3. Statistics

The goal of this analysis was to evaluate which potential mediators measured at 6 weeks mediated the effect of yoga or stretching compared to self-care on back pain dysfunction (RDQ) measured at 12 weeks. Separate analyses were conducted assessing mediators of the effect of yoga and the effect of stretching. Therefore, the cohort assessing yoga mediators included yoga and self-care participants (*n* = 118), and the cohort assessing stretching mediators included stretching and self-care participants (*n* = 114). Subset analyses including the saliva measures (cortisol and DHEA) as potential mediators were restricted to those with available saliva measurements (*n* = 82 for yoga versus self-care cohort and *n* = 76 for stretching versus self-care cohort). Cortisol and DHEA were analyzed using logarithmic transformations.

We used the classical definition of a mediator as a variable that is statistically responsible for either all or part of the effects of treatment on outcome. We initially assumed a sequential stepped process to define a mediator using a series of models addressing the following questions. (1) Is there a relationship between intervention and outcome? (2) Is there a relationship between intervention and each mediator? (3) is there a relationship between mediator and outcome? And if Steps 1–3 were confirmed, (4) is there a reduction of the intervention effect on outcome after adjusting for the mediator? For a proposed mediator variable to be considered further, both the adjusted association between 6-week mediator change and intervention group (Step 2) and the association between the 12-week RDQ outcome and the 6-week change in the mediator (Step 3) had to be statistically significant at the 0.10 level. For potential mediators meeting this criterion, we assumed that the total adjusted 12-week change in RDQ could be partitioned into the effect of the intervention on 12-week RDQ due to associated changes in the 6-week mediator variable plus the direct, unmediated effect of the intervention on 12-week RDQ. These effects were estimated using a series of linear regression models each adjusting for the following baseline covariates: age, sex, body mass index (BMI), days of back pain in the last 6 months, strenuousness of work, whether back pain travels down the leg, symptom bothersomeness, and RDQ. Standard errors were calculated using bootstrap methods [41], and bias-corrected, accelerated 95% confidence intervals [42] were calculated. To simplify the comparison of mediation effects between different mediator models, we also calculated the proportion of the total change in 12-week RDQ due to the mediator or mediators included in each model. All candidate mediators passing the significance criteria in Steps 1–3 were considered in single mediator models as well as in models including multiple mediators.

Since the interventions were hypothesized to be effective through numerous correlated mediation pathways simultaneously, we conducted exploratory analyses not requiring that each mediator had to be both related to the intervention and outcome independently (Steps 2 and 3). Specifically, we estimated the mediation effects for all linear combinations of mediators proposed in the theoretical model. We present estimates of mediated and unmediated effects from single and multiple mediator models for mediators meeting the significance criterion as well as estimates from the best possible set of all mediators, where the best possible set is defined to be the linear combination of mediators that is associated with the largest proportion of the overall reduction in intervention effect in 12-week RDQ.

### 2.4. Qualitative Questions and Analyses

At the end of the 12-week followup interview, we asked participants in the yoga and stretching groups two open-ended questions customized to their randomization group: “what effect, if any, has the [exercise or yoga] training had on you? For example, this might include your thoughts, feelings, reactions, or activities” and “what were the most important things you learned from being in those classes?” A final question inquired whether they had anything else to tell us. The short open-ended responses were coded for themes by two researchers using an iterative approach.

## 3. Results

### 3.1. Mediation Analyses

Previously, Sherman et al. [9] showed that yoga and stretching were superior to self-care. Description of the demographic characteristics, current episode of back pain, and potential mediators of the study participants are shown in Table 1. Most baseline characteristics were similar across groups. Back dysfunction was slightly worse in the yoga group, and the stretching group had slightly fewer days of back pain in the prior 6 months.

Both yoga and stretching were associated with statistically significant changes in several of the 6-week mediator variables at the 0.10 level (mediator Step 2; Table 2). Compared to usual care, yoga demonstrated improved self-efficacy (*P* = 0.010), decreased sleep disturbances due to back pain (*P* = 0.050), and increased hours of back exercise in the past week (*P* = 0.0006). Compared to usual care, stretching showed improved self-efficacy (*P* = 0.002) and increased hours of back exercise (*P* < 0.0001).

Table 3 presents estimates of the change in 12-week RDQ associated with the proposed mediator variables measured at 6 weeks after randomization (mediator Step 3). In the yoga group (compared to usual care), fear avoidance (*P* = 0.062), self-efficacy (*P* < 0.0001), conscious awareness of body (*P* = 0.027), sleep disturbances due to back pain (*P* = 0.0006), and hours of back exercise performed in the past week (*P* = 0.081) were associated with statistically significant changes in 12-week RDQ at the 0.10 level. In the stretching group (compared to usual care), fear avoidance (*P* < 0.0001), self-efficacy (*P* = 0.003), positive states of mind (*P* = 0.0064), sleep disturbances (*P* = 0.00038), and hours of back exercise (*P* = 0.040) were associated with statistically significant changes in 12-week RDQ.

The results described above identify a subset of mediator variables where both the association between the mediator and the intervention and the association between the 12-week RDQ and the 6-week change in the mediator are significant at the 0.10 level. For yoga (versus self-care), self-efficacy, sleep disturbances, and hours of exercise performed for back pain were jointly significant. For stretching (versus self-care), only self-efficacy and hours of back exercise were jointly significant.  Table 4 presents estimates of mediation effects comparing models containing each of the final mediators individually and combined for each group comparison. Estimates are also provided for a model containing the subset of all possible mediators that yielded the largest mediation effect as a proportion of the overall effect of the intervention (best overall mediator scenario).

Of the total estimated reduction in 12-week RDQ associated with yoga (−2.31, 95% CI, −3.61 to −0.93), self-efficacy accounted for 36% percent of the benefit (−0.82, 95% CI = −1.64 to −0.30), decreased sleep disturbances due to back pain accounted for 18% (−0.42, 95% CI = −1.09 to −0.03), and hours of back exercise accounted for 9% (−0.21, 95% CI = −0.91 to 0.32). The combination of these three variables accounted for 56% of the total benefit (−1.30, 95% CI = −2.44 to −0.54). The best possible subset of all potential mediator variables included self-efficacy, conscious awareness of body, psychological distress, perceived stress, sleep disturbances due to back pain, and hours of back exercise and accounted for 61% of the total decrease in 12-week RDQ due to yoga (−1.42, 95% CI = −2.47 to −0.58). Of the total estimated reduction in 12-week RDQ associated with stretching (−2.00, 95% CI = −3.37 to −0.72), self efficacy and hours of back exercise accounted for 23% (−0.47, 95% CI = −1.13 to −0.02) and 14% (−0.29, 95% CI = −1.16 to 0.72) of the benefit, respectively. Both variables together accounted for 30% of the benefit (−0.59, 95% CI = −1.58 to 0.38). The best possible subset of mediators included fear avoidance, self-efficacy, conscious awareness of body, psychological distress, positive states of mind, and hours of back exercise and accounted for 50% of the total benefit (−0.99, 95% CI = −2.38 to −0.04).

 Analyses confined to the subset of participants with saliva samples did not find that either cortisol (average awakening response) or DHEA (diurnal average) met the criteria for mediators (Tables 2 and 3). However, in the best possible combination of all mediator models, DHEA (diurnal average) contributed to the yoga model and cortisol (average awakening response) contributed to the stretching model (Table 4). 

### 3.2. Qualitative Analyses

Comments about the classes were made by 77 of the 79 interviewed participants in the yoga group who attended at least one class and 72 of the 73 interviewed participants in the stretching group who attended at least one class (Table 5). Most participants in both groups (77.2% yoga versus 68.5% stretching) mentioned that the “exercises” themselves were beneficial that they learned new or better exercises or that they had increased strength or flexibility. Some typical responses are shown below:

*It gave me good tips on doing some of the stretching exercises. (Stretching participant)*

*I've noticed, there is an increase in flexibility and strength and muscle tone. (Stretching participant)*

*It provided a safe way for strengthening and stretching my back. (Yoga participant)*

*The exercises, the stretching and strengthening I can do now. (Yoga participant)*

Other commonly reported benefits from participants in the yoga group were relaxation and stress reduction (40.5%), increased awareness (36.7%), and the importance and benefits of breathing (29%). 

Some example comments are shown below:

*I have little to no back pain anymore. It helps me relax. I sleep much better. I just feel generally stronger and healthier. My overall well-being feels better; happier, better frame of mind, not so stressed. I loved it! (Yoga participant)*

*It kind of got me back in touch with my body again and it was very calming. (Yoga participant)*

*Really positive effects! …. I would add awareness or being able to tell what I need. (Yoga participant)*

*… I can breathe easier when I feel I might be getting anxious. I have an instant position that I can maintain when I feel my back pain so it doesn't last hours as before and I am excited that I have a new tool …. (Yoga participant)*

*It's changed the way I breathe. It's changed the way I move, stand and sit. It's changed the way I hold myself like how I lift things. [I learned] how to strengthen my core muscles, how to breathe correctly, and how to hold my back and stomach. (Yoga participant)*

Participants in the yoga classes were also more likely to speak of the holistic benefits (see quotes earlier and below).

*I think its good for my back and more and I think its good for relaxing me as well as good for my muscular structure and my body core getting together with other people was good. (Yoga participant)*

*I feel stronger in my back, relief in some cases, feel relaxed and at ease, centered. (Yoga participant)*

By contrast, participants in the exercise group were more likely to mention the importance of discipline and routine (39.7%). Some examples are shown below:

*It has a really good effect, I feel better … when you get stretch out it keep, everything in sync. It makes you feel better the key is sticking to it. It's okay to skip a night, but the key is to try and do it every night. (Stretching participant)*

*Just it made me a lot more flexible to walk and things like that. It just made me more confident in my physical well being … It wasn't so much learning the exercises. It was more the idea of consistency. Having a schedule for a particular exercise was pretty important for me …. (Stretching participant)*

Roughly, 10% of participants in both groups reported increased self-efficacy. A variety of other benefits were mentioned occasionally by participants in both groups, including increased energy and better mood. Five participants in the stretching group (6.8%) thought some of the stretches were harmful, while six participants in the stretching group (8.2%) and five in the yoga group (6.3%) mentioned that the classes were not helpful or were boring. 

## 4. Discussion

Using a comprehensive set of mediator variables encompassing the categories of physical movement, cognitive appraisal, and affect and stress, we found that both self-efficacy and hours of back exercise in the prior week mediated the effects of yoga and stretching on back dysfunction. In addition, sleep disturbance had a small effect on back dysfunction in the yoga group. These variables mediated 56% of the relationship with yoga and 30% of the relationship with stretching. The strongest mediator for both treatments was self-efficacy (36% for yoga and 23% for stretching). Hours of back exercise had roughly similar mediation effects in both groups, about 10–15%. These results might be surprising because yoga and stretching had similar effects [9]. Qualitative analysis of open-ended comments suggested that the most important effect of both interventions was learning new, better exercises and relatively few comments related to self-efficacy per se. 

Few other variables explained much of the relationship between yoga and improved back dysfunction, although the qualitative analyses suggest that increased awareness and relaxation were important. Yoga is a complex multifactorial intervention with a number of potentially different therapeutic mechanisms, including physical effects of movement, benefits of breathing, and benefits of concentration. Wayne and Kaptchuk [43] described another mind-body therapy, tai chi, as a complex intervention involving at least eight components (e.g., musculoskeletal strength, flexibility and efficiency, breathing, concentration, attention, and mindfulness) that have research evidence of therapeutic effectiveness. For complex therapies like tai chi and yoga, different individuals may benefit through one or more of these components. If individuals typically benefitted from different components, we would not expect that mediator analyses would have the power to reveal this variation. In effect, we would end up having different subgroups of study participants, each of which experienced a change in different specific mediators. When only a subgroup of study participants experiences a change in a specific mediator, the statistical power to observe an overall reduction in the intervention effect due to the mediator is diminished even though the mediator effect is strong within that subgroup. 

In addition, many of the measures we selected may not have completely addressed the constructs in our model. For example, measuring body-focused awareness has been challenging because, in extant questionnaires, distinctly different processes are subsumed under this concept [44]. While most research in this area has concentrated on the harms from somatosensory amplification, some researchers use the term body awareness to refer to the salubrious ability to notice subtle bodily sensations [44]. A recent review of questionnaires purporting to measure body awareness found that none of the measures were comprehensive, and further work was needed to identify critical domains [44]. Recently, qualitative research has elucidated common themes among practitioners of body awareness practices, but at the time of our study, extant questionnaires did not measure these adequately [45]. 

 Strengths of these analyses include the comparison of two treatments with self-care, a comprehensive theory-driven selection of mediators, the measurement of mediators preceded that of outcomes which strengthens a causal interpretation, the use of qualitative results to confirm or refute findings of the formal mediation analysis, and the conduct of these analyses in the context of a large, rigorous randomized trial. 

Our mediator analyses have several limitations. Because we found no difference between yoga and stretching, we were unable to directly compare the mediators of these interventions. Furthermore, because our study population was fairly healthy and excluded individuals with moderate or severe depression, it may have been unable to detect some of the purported benefits of yoga, such as stress reduction. Finally, we did not assess mindfulness, another construct that is challenging to measure but might have accounted for some of the benefits we observed.

## 5. Conclusions

Both yoga and stretching were superior to self-care, and our mediator analyses suggest that increased participation in back exercise and self-efficacy was responsible for most of these benefits. However, these are both complex interventions and qualitative data suggesting that relaxation and increased awareness may have contributed to the benefits of yoga, while, increased discipline and routine may have contributed to the benefits of stretching. To better understand the common and unique mechanisms responsible for the benefits of yoga and exercise, future research should compare types of yoga that are more distinct from intensive stretching exercise programs through greater emphasis on meditation and breathing components.

## Figures and Tables

**Table 1:** Baseline description of yoga trial participants included in mediation analyses.

	Main cohort						Saliva cohort					
	Yoga		Stretching		Self-care		Yoga		Stretching		Self-care	
Total, *N*	78		74		40		57		51		25	
Demographics												
Age (yrs), mean (SD)	47	(9.5)	49	(10.1)	51	(8.4)	49	(8.9)	50	(9.5)	52	(7.9)
Women, *N* (%)	53	(67.9%)	47	(63.5%)	23	(57.5%)	37	(64.9%)	31	(60.8%)	15	(60.0%)
College graduate, *N* (%)	49	(62.8%)	53	(71.6%)	24	(60.0%)	34	(59.6%)	37	(72.5%)	15	(60.0%)
White, *N* (%)	68	(87.2%)	65	(87.8%)	39	(97.5%)	49	(86.0%)	43	(84.3%)	25	(100.0%)
Family income > $45,000/yr, *N*(%)	76	(97.4%)	65	(90.3%)	34	(87.2%)	55	(96.5%)	46	(92.0%)	21	(84.0%)
Lifts 20 + pounds at job, *N* (%)	16	(20.5%)	19	(25.7%)	14	(35.0%)	13	(22.8%)	10	(19.6%)	9	(36.0%)
Health behaviors												
Obese (BMI ≥30), *N* (%)	21	(26.9%)	21	(28.4%)	14	(35.0%)	13	(22.8%)	13	(25.5%)	10	(40.0%)
Drank alcohol today, *N* (%)	—	—	—	—	—	—	16	(28.1%)	18	(35.3%)	10	(40.0%)
Smoked today, *N* (%)	—	—	—	—	—	—	1	(1.8%)	3	(5.9%)	1	(4.0%)
Took medication(s) today, *N* (%)	—	—	—	—	—	—	38	(66.7%)	36	(70.6%)	19	(76.0%)
Back pain status												
Days of back pain in last 6 months, mean (SD)	146	(46.2)	132	(51.4)	148	(47.1)	146	(46.7)	134	(53.9)	155	(37.2)
Bothersomeness score, mean (SD)	4.8	(1.8)	4.4	(2.0)	4.8	(2.5)	4.8	(1.6)	4.3	(1.9)	5.0	(2.6)
Pain travels down the leg, *N* (%)	11	(14.1%)	11	(14.9%)	8	(20.0%)	10	(17.5%)	7	(13.7%)	5	(20.0%)
Back-related dysfunction												
Roland-Morris Disability Questionnaire (RDQ), mean (SD)	9.4	(4.8)	8.5	(3.7)	9.0	(4.9)	9.6	(4.7)	8.2	(3.7)	8.4	(4.9)
Potential mediators at baseline												
Positively impacts cognitive appraisal												
Fear avoidance, mean (SD)	31.5	(6.8)	32.0	(7.1)	32.0	(6.3)	31.3	(6.5)	31.9	(7.3)	32.9	(6.9)
Self-efficacy, mean (SD)	6.1	(1.9)	6.4	(1.6)	6.6	(1.6)	6.2	(1.9)	6.5	(1.4)	6.9	(1.5)
Body awareness, mean (SD)	44.1	(8.7)	41.0	(8.6)	43.9	(6.9)	44.2	(9.1)	42.4	(9.0)	43.5	(7.0)
Positively impacts affect and stress												
Psychological distress, mean (SD)	67.3	(6.9)	67.4	(7.8)	66.8	(6.7)	67.5	(6.9)	65.7	(7.8)	67.4	(6.1)
Perceived stress, mean (SD)	16.0	(5.6)	16.0	(5.3)	15.2	(5.4)	15.8	(5.8)	15.8	(5.2)	15.8	(6.0)
Positive states of mind, mean (SD)	14.8	(2.2)	15.3	(2.0)	15.5	(2.3)	14.8	(2.3)	15.3	(2.1)	15.3	(2.5)
Back pain negatively impacts sleep, *N* (%)	44	(56.4%)	42	(57.5%)	24	(60.0%)	30	(52.6%)	25	(50.0%)	14	(56.0%)
Physical activity												
Hours of back exercise in the past week, mean (SD)	0.38	(0.58)	0.27	(0.40)	0.43	(0.49)	0.36	(0.47)	0.29	(0.41)	0.41	(0.39)
Improves neuroendocrine function												
Salivary cortisol awakening response (log scale), mean (SD)	—	—	—	—	—	—	0.8	(1.3)	1.1	(1.0)	0.7	(1.3)
Salivary DHEA diurnal average (log scale), mean (SD)	—	—	—	—	—	—	−0.9	(0.7)	−1.1	(0.8)	−1.1	(0.8)

**Table 2:** Associations between 6-week change in mediator values and intervention group^#^.

	Main cohort*				Saliva cohort**			
	Change in mediator	SE	95% CI	*P* value	Change in mediator	SE	95% CI	*P* value
Yoga versus self-care								
Fear avoidance	−0.24	1.39	(−3.00, 2.52)	0.865	2.67	1.87	(−1.07, 6.41)	0.158
Self-efficacy	0.78	0.30	(0.19, 1.38)	0.010	0.63	0.37	(−0.10, 1.36)	0.090
Conscious awareness of body	1.29	1.24	(−1.16, 3.74)	0.299	−0.39	1.51	(−3.41, 2.63)	0.796
Psychological distress	−1.17	1.56	(−4.26, 1.92)	0.456	−1.42	1.89	(−5.20, 2.36)	0.455
Perceived stress	−0.22	1.02	(−2.24, 1.80)	0.828	−0.13	1.32	(−2.77, 2.51)	0.922
Positive states of mind	0.51	0.51	(−0.50, 1.52)	0.316	0.48	0.59	(−0.70, 1.66)	0.418
Improved sleep	−0.20	0.10	(−0.39, 0.00)	0.050	−0.20	0.13	(−0.45, 0.06)	0.131
Hours of back exercise	0.93	0.27	(0.41, 1.46)	0.0006	1.32	0.31	(0.70, 1.95)	0.00007
DHEA change in diurnal average (log scale)	—	—	—	—	0.19	0.13	(−0.06, 0.44)	0.139
Average cortisol awakening response (CAR)	—	—	—	—	−0.12	0.27	(−0.66, 0.42)	0.658
Stretching versus self-care								
Fear avoidance	−1.73	1.26	(−4.23, 0.77)	0.173	−0.04	1.56	(−3.16, 3.09)	0.981
Self-efficacy	0.84	0.26	(0.32, 1.36)	0.002	0.75	0.36	(0.03, 1.48)	0.042
Conscious awareness of body	0.76	1.49	(−2.18, 3.71)	0.608	−1.22	1.82	(−4.85, 2.41)	0.505
Psychological distress	−2.06	1.62	(−5.27, 1.16)	0.207	−0.17	2.07	(−4.30, 3.96)	0.934
Perceived stress	−1.34	0.86	(−3.05, 0.37)	0.122	−0.10	1.12	(−2.35, 2.14)	0.926
Positive states of mind	0.64	0.45	(−0.25, 1.52)	0.156	0.64	0.55	(−0.45, 1.73)	0.247
Improved sleep	−0.07	0.10	(−0.27, 0.12)	0.448	−0.04	0.13	(−0.30, 0.22)	0.765
Hours of back exercise	1.25	0.23	(0.79, 1.71)	0.000001	1.72	0.30	(1.13, 2.32)	0.0000002
DHEA change in diurnal average (log scale)	—	—	—	—	0.08	0.15	(−0.21, 0.37)	0.595
Average cortisol awakening response (CAR)	—	—	—	—	0.07	0.28	(−0.50, 0.64)	0.799

^#^Each mediator is an outcome variable in a model with intervention as the predictor variable and adjustment for selected baseline variables. Therefore, note that effect size comparison across rows should not be done since outcome variable is changing.

*Adjusted for baseline variables age, sex, BMI, back pain days, work type, pain travelling down the leg, bothersomeness, and RDQ. **Same adjustments as main cohort analysis with the addition of alcohol, tobacco, and medication use on the day of saliva sample collection.

**Table 3:** 12-week change in RDQ* associated with 6-week mediator values.

	Main cohort**				Saliva cohort***			
	Change in RDQ*	SE	95% CI	*P* value	Change in RDQ∗	SE	95% CI	*P* value
Yoga versus self-care								
Fear avoidance	0.10	0.05	(−0.01, 0.20)	0.062	0.11	0.06	(−0.01, 0.23)	0.069
Self-efficacy	−1.17	0.21	(−1.59, −0.74)	0.0000003	−1.23	0.27	(−1.76, −0.69)	0.00002
Conscious awareness of body	−0.13	0.06	(−0.25, −0.02)	0.027	−0.05	0.08	(−0.20, 0.10)	0.491
Psychological distress	0.05	0.05	(−0.04, 0.15)	0.287	0.02	0.06	(−0.10, 0.14)	0.700
Perceived stress	0.08	0.07	(−0.07, 0.22)	0.296	0.01	0.09	(−0.17, 0.18)	0.955
Positive states of mind	−0.12	0.15	(−0.41, 0.17)	0.418	−0.16	0.19	(−0.55, 0.22)	0.402
Improved sleep	2.51	0.70	(1.11, 3.90)	0.00055	2.26	0.84	(0.58, 3.94)	0.009
Hours of back exercise	−0.46	0.263	(−0.99, 0.06)	0.081	−0.59	0.32	(−1.23, 0.05)	0.070
DHEA change in diurnal average (log scale)	—	—	—	—	−0.60	0.89	(−2.38, 1.18)	0.502
Average cortisol awakening response	—	—	—	—	−0.88	0.41	(−1.70, −0.06)	0.035
Stretching versus self-care								
Fear avoidance	0.20	0.05	(0.10, 0.30)	0.000094	0.26	0.07	( 0.13, 0.39)	0.00017
Self-efficacy	−0.72	0.24	(−1.19, −0.25)	0.003	−0.75	0.29	(−1.32, −0.17)	0.012
Conscious awareness of body	−0.05	0.05	(−0.15, 0.04)	0.240	0.01	0.06	(−0.12, 0.13)	0.921
Psychological distress	0.05	0.04	(−0.04, 0.13)	0.252	0.04	0.06	(−0.08, 0.14)	0.528
Perceived stress	0.00	0.08	(−0.16, 0.15)	0.979	−0.03	0.10	(−0.23, 0.18)	0.794
Positive states of mind	−0.41	0.15	(−0.70, −0.12)	0.0064	−0.43	0.20	(−0.83, −0.03)	0.035
Improved sleep	2.43	0.66	(1.12, 3.74)	0.00038	2.55	0.80	(0.95, 4.16)	0.002
Hours of back exercise	−0.53	0.26	(−1.04, −0.03)	0.040	−0.31	0.31	(−0.92, 0.30)	0.307
DHEA change in diurnal average (log scale)	—	—	—	—	1.20	0.77	(−0.33, 2.73)	0.122
Average cortisol awakening response	—	—	—	—	−0.71	0.39	(−1.49, 0.07)	0.073

*RDQ: Roland Morris Disability Questionnaire.

**Adjusted for baseline variables age, sex, BMI, back pain days, work type, pain traveling down the leg, bothersomeness, and RDQ.

***Same adjustments as main cohort analysis with the addition of alcohol, tobacco and medication use on the day of saliva sample collection.

**Table 4:** Assessment of the reduction of the intervention effect on twelve-week change in RDQ* associated with mediator(s).

	Total intervention effect	Remaining effect after mediator(s)	Effect reduced by mediator(s)		% Reduction due to mediator(s)
			Reduction (95% CI)		
(I) Main cohort					

Yoga versus self-care					
Self-efficacy only (A)	−2.31	−1.48	−0.82	(−1.64, −0.30)	35.7%
Improved sleep only (B)		−1.88	−0.42	(−1.09, −0.03)	18.3%
Hours of back exercise only (C)		−2.09	−0.21	(−0.91, 0.32)	9.2%
All A + B + C in the model		−1.01	−1.30	(−2.44, −0.54)	56.4%
Best overall mediator scenario^1^		−0.89	−1.42	(−2.47, −0.58)	61.3%
Stretching versus self-care					
Self-efficacy only	−2.00	−1.54	−0.47	(−1.13, −0.02)	23.3%
Hours of back exercise only		−1.72	−0.29	(−1.16, 0.72)	14.3%
A + B in the model		−1.41	−0.59	(−1.58, 0.38)	29.6%
Best overall mediator scenario^2^		−1.01	−0.99	(−2.38, −0.04)	49.6%

(II) Saliva cohort					

Yoga versus self-care					
Self-efficacy only	−2.48	−1.78	−0.70	(−1.85, −0.09)	28.2%
Hours of back exercise only		−2.16	−0.32	(−1.24, 0.57)	13.0%
A + B in the model		−1.35	−1.13	(−2.60, −0.14)	45.5%
Best overall mediator scenario^3^		−0.61	−1.87	(−3.48, −0.49)	75.5%
Stretching versus Self-Care					
Self-efficacy only	−2.08	−1.62	−0.46	(−1.40, 0.01)	21.9%
Best overall mediator scenario^4^		−1.34	−0.74	(−2.08, 0.23)	35.4%

*RDQ: Roland-Morris Disability Questionnaire.

Model framework: each line is a separate model assessing the reduction in intervention effect after including mediator(s) in the model. Both the estimated total intervention (without any mediators) and mediator(s) models adjust for baseline variables age, sex, BMI, back pain days, work type, pain travelling down the leg, bothersomeness, and RDQ. Saliva cohort has the same baseline adjustments as the main cohort analysis with the addition adjustment of alcohol, tobacco, and medication use on the day of saliva sample collection.

Best overall mediator scenarios include the following mediators:

^1^Self-efficacy, conscious awareness of body, psychological distress, perceived stress, sleep disturbance, and hours of back exercise.

^2^Fear, self-efficacy, conscious awareness of body, psychological distress, positive states of mind, and hours of back exercise.

^3^Self-efficacy, Psychological Distress, perceived stress scale, sleep disturbance, hours of back exercise, and DHEA diurnal average (log scale).

^4^Self-efficacy, conscious awareness of body, perceived stress scale, positive states of mind, and cortisol awakening response.

**Table 5:** Frequency of qualitative themes by treatment group.

	Yoga		Stretching	
	*N*	%	*N*	%
Theme				
Physical practice of exercise and learning about exercise	61	77.2%	50	68.5%
Relaxation, stress reduction	32	40.5%	3	4.1%
Increases awareness	29	36.7%	6	8.2%
Importance and benefits of breathing	23	29.1%	0	0.00%
Importance of discipline and routine	12	15.2%	29	39.7%
Self-efficacy	10	12.7%	8	11.0%
